# Rapid-Onset Obesity With Hypothalamic Dysfunction, Hypoventilation, Autonomic Dysregulation, and Neuroendocrine Tumor (ROHHADNET) Syndrome: A Case Report

**DOI:** 10.7759/cureus.41413

**Published:** 2023-07-05

**Authors:** Mohammed Aldirawi, Lemis Yavuz, Yousra Ghoweba, Saril Mohamedali, Nidheesh Chencheri, Nandu Thalange

**Affiliations:** 1 Pediatrics, Al Jalila Children’s Specialty Hospital, Dubai, ARE; 2 Emergency, Al Jalila Children’s Specialty Hospital, Dubai, ARE; 3 Pediatric Neurology, Al Jalila Children’s Specialty Hospital, Dubai, ARE; 4 Medicine, Mohammed Bin Rashid University, Dubai, ARE; 5 Endocrinology, Al Jalila Children’s Specialty Hospital, Dubai, ARE

**Keywords:** neural crest tumor, oligoclonal bands, autonomic dysregulation, hypothalamic dysfunction, rohhadnet, rapid-onset obesity

## Abstract

Here, we report the case of a rare and complex disorder, rapid-onset obesity with hypothalamic dysfunction, hypoventilation, autonomic dysregulation, and neuroendocrine tumor (ROHHADNET) syndrome, in a three-year-old girl with no significant medical history. This is the first such case reported from the UAE. ROHHADNET is a rare disorder of respiratory control and autonomic nervous system regulation with endocrine abnormalities. It typically presents in children older than 18 months with rapid weight gain. This is a challenging diagnosis as there is no clear diagnostic test, and treatment is essentially supportive. This report describes a case of ROHHADNET syndrome in a previously well child who presented with rapid weight gain followed by ophthalmoplegia, dysphagia, electrolyte disturbance, and other comorbidities. The paper outlines in detail the clinical course, investigations, and management of ROHHADNET syndrome. Cerebrospinal fluid analysis revealed oligoclonal bands, which have been reported in only two other cases of ROHHADNET syndrome. Our goal in reporting this case is to increase awareness of this condition among clinicians to facilitate early diagnosis and timely management.

## Introduction

Rapid-onset obesity with hypoventilation, hypothalamic, autonomic dysregulation, and neuroendocrine tumor (ROHHADNET) syndrome is a rare and frequently fatal polymorphic disorder involving multiple systems. It was first reported in 1965, and since then, only 160 cases have been documented [1]. Although the exact cause remains unknown, some evidence suggests a pathological autoimmune response, in some cases, triggered by a neural crest tumor [2-6]. The syndrome may be mistaken for other forms of hypothalamic obesity which result in abnormal hypothalamic-pituitary function, such as Prader-Willi syndrome [7].

Rapid-onset obesity with hypothalamic dysfunction, hypoventilation, and autonomic dysregulation (ROHHAD) may rapidly progress to central hypoventilation and death. This highlights the critical importance of timely diagnosis and management in such patients, particularly the provision of overnight respiratory support to minimize morbidity and mortality [8,9].

## Case presentation

**Figure 1:**
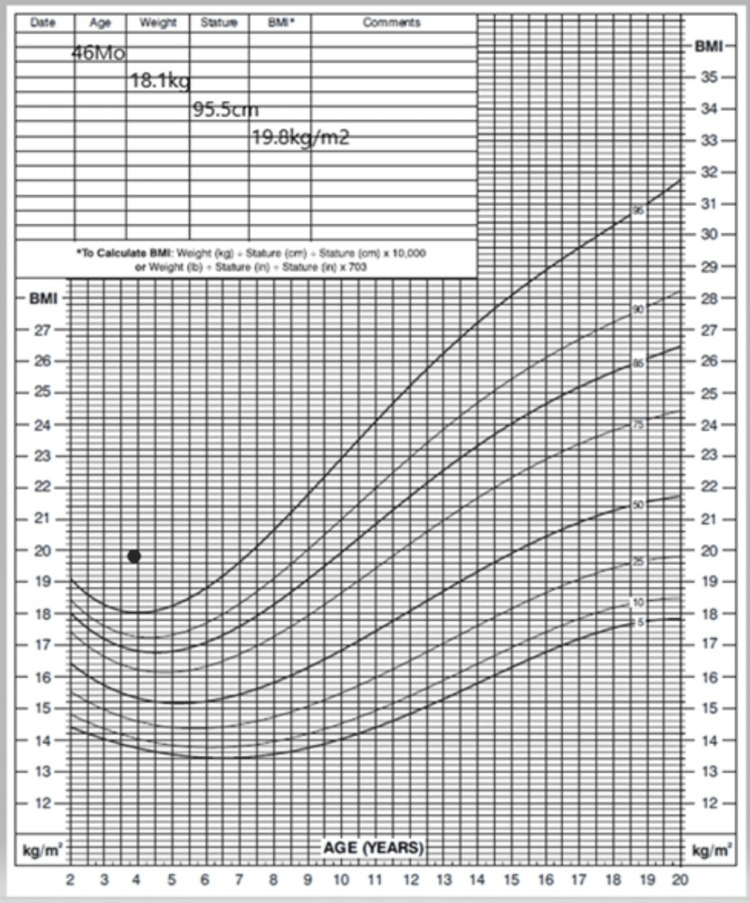
Body mass index.

A three-year-old previously healthy girl presented to our hospital with a two-week history of ophthalmoplegia and ataxia. This was associated with mood changes, social withdrawal, markedly increased appetite, and rapid weight gain over three months (Figure 1). Her parents had initially attributed her rapid weight gain and behavioral symptoms to home quarantine, with a consequent lack of physical activity during the COVID-19 pandemic. She had also experienced new-onset dysphagia, polyuria, and nocturnal enuresis. Her weight had increased from 14.0 kg (25th percentile) to 18.1 kg (85.9th percentile) at presentation, corresponding to a body mass index (BMI) of 19.8 kg/m² (98.9th percentile) (Figure 1). She had no prior medical history and was born to healthy, unrelated parents. Her antenatal and birth history was unremarkable. She had normal growth and development until symptom onset. Her family history was negative for any neurological or genetic disorders.

At presentation, the child was alert and oriented but had impaired horizontal gaze and normal vertical gaze. The pupils were 4 mm and reacted to 3 mm to light, both on direct and consensual testing. The pupils were round and centered in the middle of the iris. She had a complete range of extraocular movements. There was no ptosis. She did not exhibit nystagmus or facial palsy. There was no bulbar weakness or dysfunction, and the uvula was on the midline. The tongue did not deviate and was not atrophic. Hearing and balance were intact. There was no weakness of the Trapezii or sternocleidomastoid muscle. Upper and lower limbs had normal power, tone, and reflexes. Her gait was broad-based and she walked cautiously, partly due to her horizontal gaze difficulty. No dysarthria or cerebellar signs were detected. Apart from obesity, the rest of the physical examination was normal.

Blood tests on admission are summarized in Table 1. The results showed hyponatremia, striking hyperprolactinemia, elevated adrenocorticotropic hormone (ACTH), and slightly elevated morning cortisol in addition to microcytic, hypochromic anemia. Luteinizing hormone (LH), follicle-stimulating hormone (FSH), growth hormone (GH), and insulin-like growth factor-1 (IGF-1) were all within the normal range.

**Table 1:** Blood, urine, and cerebrospinal fluid test results. MCV = mean corpuscular volume; MCH = mean corpuscular hemoglobin; WBC = white blood cell; AST = aspartate transaminase; ALT = alanine transaminase; TSH = thyroid-stimulating hormone; ACTH = adrenocorticotropic hormone; LH = luteinizing hormone; FSH = follicle-stimulating hormone; GH = growth hormone; IGF-1 = insulin-like growth factor 1; CK = creatine kinase; anti-MOG = anti-myelin oligodendrocyte glycoprotein; NMO = neuromyelitis optica; AQP4 = aquaporin‐4

Investigations	Values
Complete blood count	
Hemoglobin	11.3 g/dL
MCV	60.7 fL
MCH	19.9 pg
WBC	15.15 × 10^3^/µL
Biochemistry	
Osmolality	283 mOsmol/kg (normal: 275–295)
Glucose	79 mg/dL
Sodium	128 mmol/L (normal: 136–145)
Total Bilirubin	0.22 mg/dL
AST	44 U/L
ALT	29 U/L
Endocrinology	
TSH	3.75 mIU/L (normal: 0.7–5.9)
T4	17.5 pmol/L (normal: 12.3–22.8)
Prolactin	178.4 ng/mL (normal: 4.8–23.3)
ACTH	74 pg/mL (normal: 7.2–63.3)
Cortisol AM	11.5 µg/dL (normal: 3.7–9.4)
LH	<0.1 mIU/mL
FSH	0.3 mIU/mL (normal: 0.2–11.1)
GH	1.88 ng/mL (normal: 0–10)
IGF-1	67 ng/mL (normal: 34.2–155)
Specific blood tests	
CK	67 U/L (normal: 0–149)
Alpha-fetoprotein	2.2 ng/mL (normal: 0–8.3)
Neuron-specific enolase	16.3 ng/mL (normal: 0–12.5)
Paraneoplastic autoantibodies	Negative
Anti-neuronal nuclear antibody (I, II, III)	Negative
Anti-glial nuclear antibody (I)	Negative
Purkinje cell cytoplasmic antibody (I, II, III)	Negative
Normetanephrine	65.3 pg/mL
Metanephrines	46.5 pg/mL (normal: 0–88.0)
Urine investigations	
Osmolality	260 mOsmol/kg (normal: 50–1400)
Specific gravity	<1.005
Sodium	26 mmol/L
VMA/Creatinine	5.7 mg/g creat (normal: 0–11)
HVA/Creatinine	14 mg/g creat (normal: 0–22)
Cerebrospinal fluid investigations	
Glucose	59 mg/dL
Protein	16 mg/dL
WBC	4
Neutrophils	0%
Lymphocytes	100%
Viral panel	Negative
Culture	Negative
Lactate	1.9 mmol/L (normal: 1.1–2.8)
Oligoclonal bands	Positive for oligoclonal bands
Anti-MOG antibodies	Negative
NMO/AQP4 IgG	Negative

Upon admission, the patient was evaluated by a multidisciplinary team that included endocrinology, neurology, gastroenterology, pulmonology, cardiology, oncology, pediatric surgery, and speech pathology services.

After admission, polyuria (urine output >4 mL/kg/hour) and nocturnal enuresis with dilute urine (specific gravity <1.010, urine osmolality <300) were noted. Despite hyponatremia, it was felt to be consistent with arginine vasopressin deficiency (previously known as central diabetes insipidus), and she responded well to desmopressin, which normalized her polyuria and hydration [10].

The patient presented initially with oculomotor apraxia along with unsteady gait that progressed over a few days to include motor apraxia involving her upper limbs, and, subsequently, increasing weakness of palmar grasp. Physiotherapy was commenced. Along with her progressive neurological deterioration, she had worsening behavior with marked emotional lability. A lumbar puncture was done, and cerebrospinal fluid (CSF) analysis was positive for oligoclonal bands but otherwise unremarkable.

In view of the known high risk of central apnea, polysomnography was performed and showed moderate sleep apnea with an apnea-hypopnea index of 10 per hour; however, no significant desaturations or hypoventilation occurred during the study. We recommended overnight bilevel-positive airway pressure and oxygen saturation monitoring at home due to her risk of central hypoventilation; however, the parents refused.

The patient had difficulty chewing and swallowing solid food upon presentation and progressed dysphagia for liquids. Her modified barium swallow study showed moderate dysphagia with uncoordinated swallowing. Given her high aspiration risk, she was limited to pureed food and fluids through a nasogastric tube and, later, via gastrostomy.

**Figure 2:**
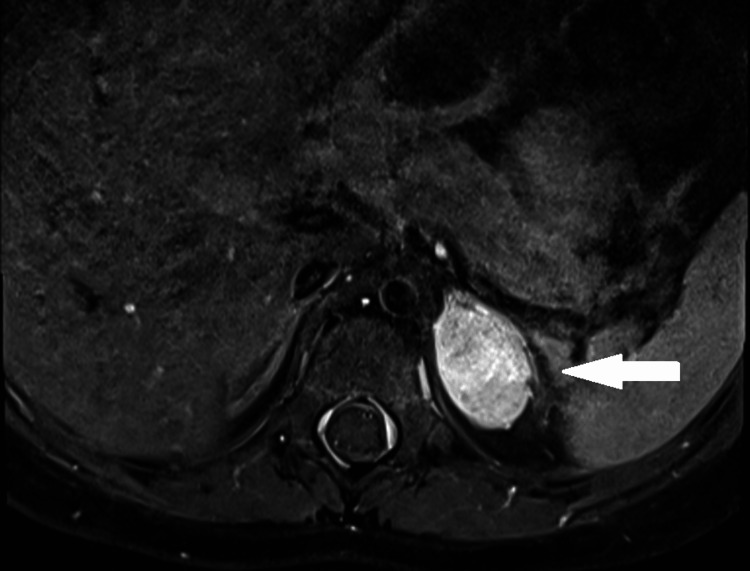
MRI: Left suprarenal mass showing contrast enhancement of her left adrenal mass, which was confirmed on histology to be a ganglioneuroma.

Given her horizontal gaze palsy and progressive dysphagia, she underwent a whole brain and spine MRI which identified a mass in the left suprarenal region (Figure 2). CT of the chest and abdomen confirmed a left adrenal mass measuring approximately 1.6 × 2.6 × 2.4 cm.

At laparoscopy, a 4.5 × 3.0 × 2.0 cm smooth, encapsulated adrenal mass was found and completely resected. Histology of the tumor showed Schwannian stroma and ganglion cells, typical of a ganglioneuroma. There were multiple foci of calcification along with clusters of lymphocytic infiltration, without any noted neuroblast or neuropil tissue.

Molecular analysis for *PHOX2B *was performed to rule out congenital central hypoventilation syndrome (CCHS). With the rapid development of obesity, disordered water balance (hyponatremia), hyperprolactinemia, abnormal polysomnography, and the absence of *PHOX2B *mutations, our patient’s diagnosis was confirmed to be ROHHADNET.

She received intravenous immunoglobulin (IVIG) 2 g/kg over two days, and we planned to proceed to rituximab and cyclophosphamide if she proved refractory to IVIG therapy. At follow-up, she had greatly improved behavior and swallowing. Unfortunately, the parents subsequently defaulted from follow-up, and she died in her sleep six months later.

## Discussion

We describe the case of a three-year-old girl who presented with classical signs and symptoms of ROHHAD. The investigation confirmed the presence of a left adrenal neuroendocrine tumor and the absence of *PHOX2B* mutation, confirming the full ROHHADNET syndrome. Although the diagnosis of this syndrome is often delayed, in our case, it was suspected at the initial presentation. Management is very challenging and requires a multidisciplinary approach involving pulmonology, endocrine, autonomic medicine, oncology, psychiatry, surgery, ENT, cardiology, psychology, and dietetics [9,11].

Our differential diagnosis included endocrinological etiologies such as hypercortisolism and hypothyroidism, which were excluded on initial tests. The finding of hyponatremia coupled with polyuria was contradictory, but the patient responded extremely well to DDAVP therapy, with the restoration of hydration and consequent improved well-being. The co-occurrence of arginine vasopressin deficiency and cerebral salt wasting is a potential explanation for this anomalous presentation. Striking hyperprolactinemia was noted at presentation and is characteristic of ROHHAD, indicating a more generalized hypothalamic dysfunction. Dysnatremia and hyperprolactinemia were seen in 97% and 96% of ROHHAD cases, respectively [9]. Her 9 am ACTH and cortisol, although trivially elevated, were not consistent with Cushing’s disease, and were most likely due to stress.

One of the other differential diagnoses was CCHS which was suspected based on the clinical presentation of ventilatory dysfunction, especially during sleep, without evidence of primary respiratory, cardiac, or neurologic disorders [12]. The diagnosis of CCHS can be confirmed by polysomnography findings and genetic testing revealing a *PHOX2B *mutation [12].

Although an underlying genetic component has been proposed for the pathogenesis of ROHHAD due to its similarity with CCHS, extensive screening and chromosomal analysis failed to identify any genetic mutation [3,4,13]. Nevertheless, genetic testing is recommended for all suspected cases to rule out a *PHOX2B *mutation and genetic obesity syndromes [14].

Prader-Willi syndrome is another differential, a rare, complex, multisystem genetic disorder recognized as the most commonly known genetic cause of life-threatening obesity in humans. The cardinal clinical features include severe infantile hypotonia, hyperphagia with the onset of obesity during early childhood if not controlled, developmental delay with learning and behavioral problems, short stature with small hands/feet, and hypogonadism/hypogenitalism due to growth hormone and other endocrine deficiencies [15].

One of the early factors in suspecting ROHHAD syndrome in a patient is rapid weight gain with increased BMI. The standard measure of obesity for children above than age of two years is a BMI ≥95th percentile for age and sex, as in our case [16,17]. The criteria for diagnosis are summarized in Table 2.

**Table 2:** Diagnostic criteria for ROHHADNET syndrome. ROHHADNET = rapid-onset obesity with hypothalamic dysfunction, hypoventilation, autonomic dysregulation, and neuroendocrine tumor

	Diagnostic Criteria for ROHHADNET	Our patient
1	Rapid-onset obesity starting after the age of 18 months	Yes
2	Evidence of hypothalamic dysfunction, as defined by at least one of the following findings:	2/6
2A	Hyperprolactinemia	Yes
2B	Central hypothyroidism	No
2C	Disordered water balance	Yes
2D	Growth hormone deficiency	No
2E	Corticotrophin deficiency	No
2F	Delayed or precocious puberty	No
3	Alveolar hypoventilation during sleep starting after the age of 18 months	Yes
4	Features of autonomic dysregulation	Yes
Rapid development of obesity was noticed at 3½ years of age, with BMI rising from the 25th to the 95th centile in just three months. Disordered water balance (hyponatremia) and hyperprolactinemia were identified at the initial presentation, Cyanotic episodes attributed to central hypoventilation were noticed four months later		

In a systematic review of ROHHADNET in 2018 by Lee et al., the most common presentation of patients with ROHHAD/NET was rapid obesity and hypothalamic dysfunction (83%), followed by hypoventilation (75%). Ocular symptoms were reported in 25% of cases [9].

At the time of diagnosis, a significant proportion of patients (42%) require respiratory support and mechanical ventilation [9]. Given our patient’s abnormal polysomnography findings along with episodes of cyanosis, central hypoventilation syndrome was diagnosed.

Our patient presented with oculomotor apraxia and a left adrenal mass. Opsoclonus-myoclonus syndrome (OMS) was excluded by the absence of clinical findings. Furthermore, tumor histology showed ganglioneuroma. OMS is typically seen with less differentiated neuroblastomas or ganglioneuroblastomas. Plasma metanephrines and normetanephrines and urine catecholamines were normal.

Lee et al. reported that the most common neuroendocrine tumors in ROHHADNET were ganglioneuromas (60%), as in our case. Although the lesions usually presented as intra-abdominal masses, two cases with mediastinal masses were reported. The characteristic findings on histology are Schwannian stroma and ganglion cells [2-6,9]. They are benign, differentiated tumors and complete resection is the preferred modality of treatment [18].

Behavioral change is a common manifestation of cognitive dysfunction (60% of cases), with symptoms such as mood changes, fatigue, social withdrawal, poor school performance, and intellectual disability, which were also noted in our patient. Other manifestations include seizures, altered consciousness, sleep disturbance, and developmental delay [9].

Although, the pathogenesis of ROHHAD has not been yet identified, an autoimmune origin is suspected [14]. The finding of oligoclonal bands in our patient and two previously reported cases support an immune origin for ROHHAD [14,19,20]. This is supported by the patient’s partial, albeit significant, clinical improvement with IVIG treatment [21].

Immunosuppressive treatment with high-dose cyclophosphamide has been reported to have positive effects on BMI stability and neuropsychological function in two cases [13,19]. Immune treatments were previously employed in six reported patients, which included glucocorticoids, IVIG, cyclophosphamide, and rituximab, with variable but generally positive responses [14].

The management of ROHHADNET syndrome requires a multidisciplinary approach (Table 3). The aim is to manage each aspect of the disease to improve quality of life and life expectancy. This necessitates the early involvement of a multidisciplinary team to manage all aspects of this polymorphic disorder. Due to the high risk of central hypoventilation, overnight polysomnography is essential for the detection of obstructive and/or central sleep apnea. Cardiac evaluation for autonomic neuropathy is needed. Hypertension requiring antihypertensive medication is common, and a pacemaker may be required to manage dysrhythmias. Neurological and ophthalmological manifestations, such as ophthalmoplegia and seizures, necessitate early involvement of these specialties [14].

**Table 3:** Multidisciplinary team in treating ROHHADNET. ROHHADNET = rapid-onset obesity with hypothalamic dysfunction, hypoventilation, autonomic dysregulation, and neuroendocrine tumor

Investigation/Screening	Therapeutic options
Rapid obesity	
Initial clinical evaluation, brain MRI, complete endocrine workup to exclude other differential diagnosis of precautious puberty, and evaluation of metabolic disturbance/year	Body mass index stabilization using strict calorie intake and regular exercise. Antidiabetic drugs and antilipid drugs
Hypothalamic dysfunction	
Hormonal investigation/Year for hypothyroidism, hyperprolactinemia, growth hormone deficiency, puberty delay, and adrenalin insufficiency	Specific hormonal substitution
Hypoventilation	
Polysomnography with nocturnal gas exchange/year if negative in the first five years, and prevention of respiratory infection	Artificial ventilation and influenza vaccine/year
Autonomic dysregulation	
ECG, echocardiography, 72-hour Holter/year, blood pressure/three months, gastroenterology screening/year for celiac disease, food intolerance and transient dysregulation, and ophthalmologic evaluation/year	Cardiac pacemaker, antihypertensive drugs, gluten-free diet, lactose-free diet, and drugs for transient control
Neural Tumor	
Screening to detect neuroendocrine tumors, and chest and abdominal MRI/year	In case of neuroendocrine tumor: staging and treatment
Neurologic Impact	
Electroencephalography in case of seizure, and evaluation for behavioral disturbances.	Antiepileptic drugs and antipsychotic drugs
Genetic considerations	
Exclude a *PHOX2B *mutation and genetic obesity	

ROHHAD(NET) syndrome is a life-threatening condition with high mortality and few long-term survivors [22]. A systematic review by Julie et al. found the median age of death to be 4.6 years and that two of six patients died due to sudden death [14]. Death at a young age may be due to delay in diagnosis, severe initial presentation, or, as in our case, parents not adhering to medical advice or follow-up.

## Conclusions

ROHHAD syndrome is a rare and potentially fatal disease that requires timely diagnosis and management by a multidisciplinary team. The finding of CSF oligoclonal bands in ROHHAD syndrome, as previously noted in two other cases, is further evidence of an immune etiology for this condition and may point the way to the use of immune therapies as a strategy for improving the prognosis of this very severe condition.
